# Remodeling of the Actin/Spectrin Membrane-associated Periodic Skeleton, Growth Cone Collapse and F-Actin Decrease during Axonal Degeneration

**DOI:** 10.1038/s41598-018-21232-0

**Published:** 2018-02-14

**Authors:** Nicolas Unsain, Martin D. Bordenave, Gaby F. Martinez, Sami Jalil, Catalina von Bilderling, Federico M. Barabas, Luciano A. Masullo, Aaron D. Johnstone, Philip A. Barker, Mariano Bisbal, Fernando D. Stefani, Alfredo O. Cáceres

**Affiliations:** 1 Instituto de Investigación Médica Mercedes y Martín Ferreyra, INIMEC-Consejo Nacional de Investigaciones Científicas y Técnicas (CONICET), UNC, Friuli, 2434 – 5016 Córdoba, Argentina; 2 Universidad Nacional de Córdoba, Córdoba, Argentina; 3 Centro de Investigaciones en Bionanociencias (CIBION)-CONICET, Buenos Aires, Argentina; 4 Departamento de Física, Facultad de Ciencias Exactas y Naturales, Universidad de Buenos Aires, Buenos Aires, Argentina; 5 Department of Biology, University of British Columbia, Kelowna, BC Canada; 6 Montreal Neurological Institute, McGill University, Montreal, Canada; 7 Instituto Universitario Ciencias Biomédicas de Córdoba (IUCBC), Córdoba, Argentina

## Abstract

Axonal degeneration occurs in the developing nervous system for the appropriate establishment of mature circuits, and is also a hallmark of diverse neurodegenerative diseases. Despite recent interest in the field, little is known about the changes (and possible role) of the cytoskeleton during axonal degeneration. We studied the actin cytoskeleton in an *in vitro* model of developmental pruning induced by trophic factor withdrawal (TFW). We found that F-actin decrease and growth cone collapse (GCC) occur early after TFW; however, treatments that prevent axonal fragmentation failed to prevent GCC, suggesting independent pathways. Using super-resolution (STED) microscopy we found that the axonal actin/spectrin membrane-associated periodic skeleton (MPS) abundance and organization drop shortly after deprivation, remaining low until fragmentation. Fragmented axons lack MPS (while maintaining microtubules) and acute pharmacological treatments that stabilize actin filaments prevent MPS loss and protect from axonal fragmentation, suggesting that MPS destruction is required for axon fragmentation to proceed.

## Introduction

Axonal degeneration during development is a critical event that distinguishes neurons that successfully compete for activity- or target-derived trophic factors from those that do not^1^. Various novel regulatory mechanisms that impinge on axonal degeneration have been recently revealed. However, knowledge on how these cascades impair the structure and function of an axonal segment is still rudimentary.

Actin cytoskeleton regulators such as ROCK^2^, cofilin^3,4^ or spectraplakin^5^ have been reported to have a role in diverse modes of axonal degeneration. Also, enzymes related to actin dynamics were found to be highly regulated in injured CNS axons as shown by unbiased proteomics of soluble axoplasm^6^ and drugs that promote F-actin disassembly activate pro-degenerative cascades^7^. Altogether, this evidence suggests that the actin cytoskeleton is modified during degeneration with a negative impact on axonal stability. However, a detailed characterization of the time course, location and type of actin structures altered during axonal degeneration is still lacking.

Fluorescence nanoscopy recently unveiled an actin/spectrin membrane-associated periodic skeleton (MPS) that is ubiquitously present in mature axons from all neuronal types evaluated so far^8–10^. Fluorescence nanoscopy reveals the MPS alternating periodic distribution of actin and spectrin, with a spacing of ~190 nm. Despite recent characterizations of its components and its response to actin depolymerizing drugs^8,11^ or genetic manipulations^11–13^, a physiological condition altering this structure (i.e. abundance, organization, distribution) has not yet been reported.

We now report fast and profound remodeling of the actin cytoskeleton across different domains of dorsal root ganglion (DRG) axons during degeneration *in vitro* induced by trophic factor withdrawal (TFW), including early and massive growth cone collapse (GCC). The MPS shows a significant decrease in abundance during most of the degeneration period, and is fully dismantled in fragmenting axons. Interestingly, an F-actin stabilizer (cucurbitacin E) when applied acutely before fragmentation prevents both MPS loss and axonal fragmentation.

## Results

### Growth Cone Collapse and F-actin Depolymerization are Early Features of Axonal Degeneration

**Figure 1:**
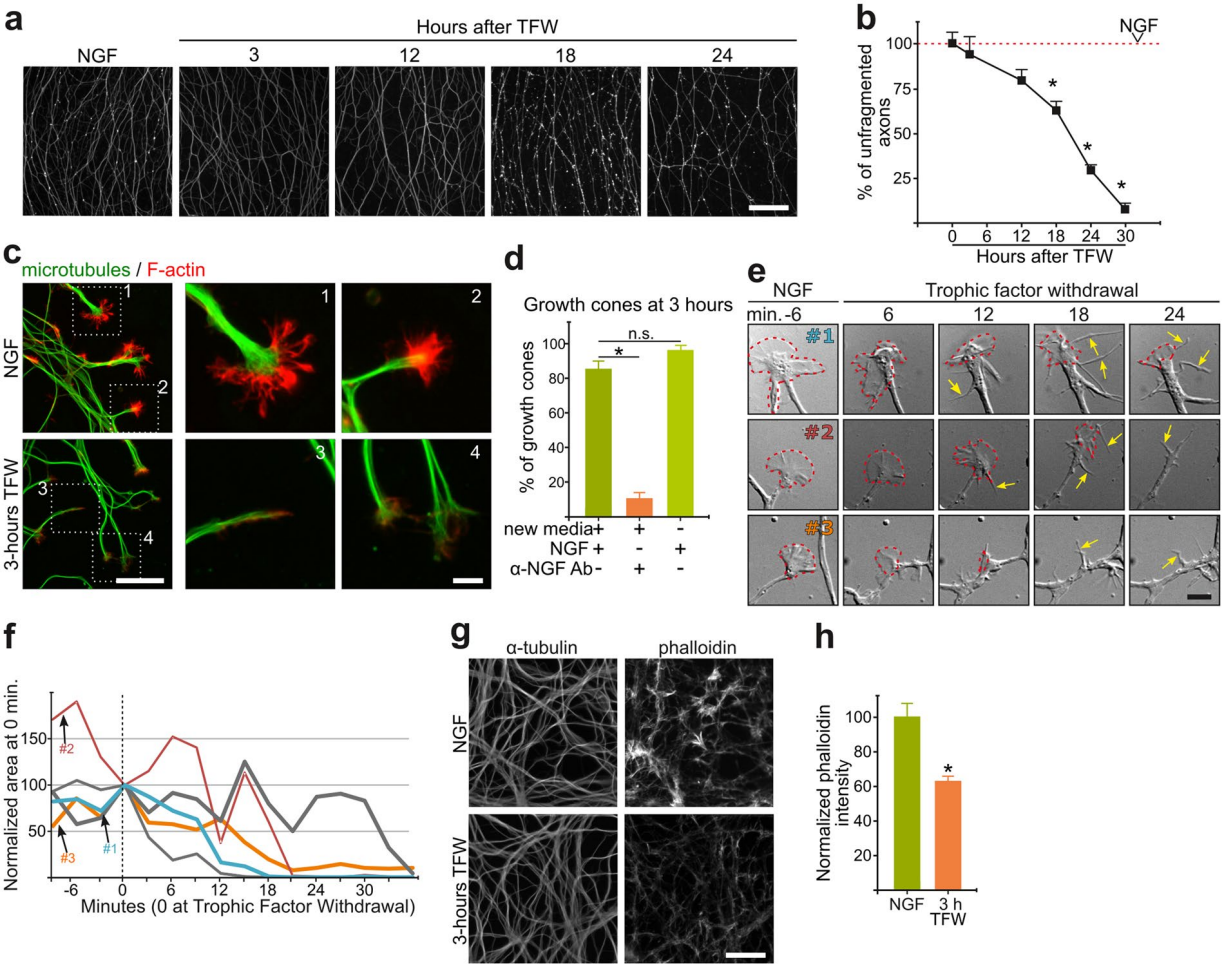
Actin remodeling and growth cone collapse are early responses to NGF withdrawal. (**a**,**b**) Representative images (**a**) and quantification (**b**) of βIII-tubulin-stained axons in a time course of axonal degeneration induced by trophic factor withdrawal (TFW). Scale bar: 100 μm. (**c**) Representative pictures of axon tips stained for α-tubulin (green) and F-actin (phalloidin, red) in control and deprived explants. Inserts 1 and 2 show normal, expanded growth cones and inserts 3 and 4 show axon tips whose growth cones are considered collapsed for lacking an F-actin-enriched lamellipodia or multiple filopodia. Scale bar: 10 μm (left) and 2 μm (right). (**d**) Quantification of the proportion of growth cones in explants maintained in NGF or deprived for 3 hours. The third is a control consisting in removing and re-adding the maintenance media. (**e**) Representative axonal tips followed in time-lapse by differential interference contrast microscopy before and after trophic factor withdrawal. The dotted line marks the growth cone area. Arrow-heads point to newly added filopodia. Scale bar: 1 μm. (**f**) Plot of growth cone areas of different axonal tips (#1, #2 and #3 shown in e) at different time points before and after TFW. (**g**,**h**) Representative images (**g**) and quantification (**h**) of the intensity of phalloidin staining in axonal shafts in control and 3-hour deprived explants. Scale bar: 50 μm. Data are represented as mean ± SEM.

To characterize changes in the actin cytoskeleton during axonal degeneration in a physiologically inspired model, we used embryonic DRG explants to recapitulate developmental pruning triggered by TFW. In this setting, nerve growth factor (NGF) supports survival and axonal growth for 3 days and subsequent NGF withdrawal induces axons to undergo a characteristic degeneration process that culminates with axonal fragmentation and detachment within approximately 24 hours after TFW (Fig. 1a,b). Growth cones are F-actin-rich structures at the tip of growing axons whose movements and dynamic morphology greatly depends on regulated actin polymerization/depolymerization cycles^14^ and we therefore followed growth cone morphology after TFW as a read out for changes in actin dynamics. GCC is a normal event during development by which extending axons are repelled from growing into inhibitory environments^15^. Surprisingly, although axons fragment by 24 hours of TFW, we found that GCC is substantial by 3 hours (Fig. 1c,d). The growth cone is a reactive structure, and hence its changes could simply reflect a response to experimental manipulations unrelated to degeneration. We thus evaluated if changing media could induce transient GCC: intact cultures were compared with cultures whose media was taken out and added back, and found no effect in GCC (Fig. 1d, third bar). Time-lapse microscopy of axonal tips shows that GCC start almost immediately after TFW, with growth cone area reaching its minimum within 30 minutes (Fig. 1e,f) and was accompanied by increased filopodia extensions and retractions close to axonal tips (Fig. 1e arrows), which ceased by 1 hour of NGF deprivation. TFW also induced a widespread decrease in phalloidin stained F-actin (Fig. 1g,h), suggesting a global response of the actin cytoskeleton to NGF deprivation.

**Figure 2:**
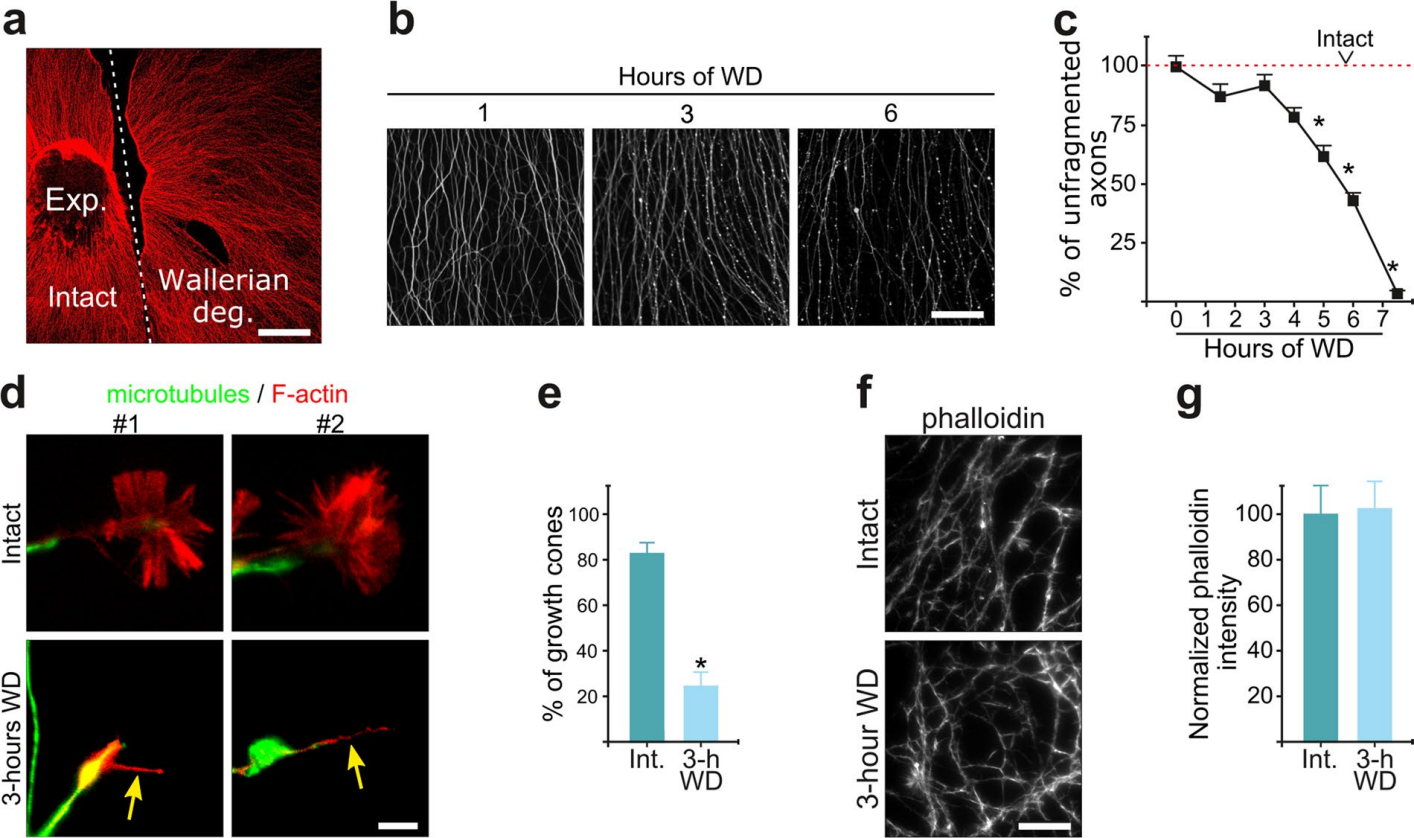
Actin remodeling and growth cone collapse are early responses to Wallerian degeneration. (**a**) Low magnification micrograph of a DRG explant (Exp.) with a transection (segmented line) to induce Wallerian degeneration (right side of the transection). (**b**,**c**) Representative images (**b**) and quantification (**c**) of the time course of axonal fragmentation during Wallerian degeneration (WD) induced by axon transection. Scale bar: 100 μm. (**d**) Representative images of axon tips stained for alpha-tubulin (green) and F-actin (phalloidin, red) in control and transected explants. Scale bar: 2 μm. (**e**) Quantification of the proportion of growth cones in intact explants and 3 hour after axonal transection. (**f**,**g**) Representative images (**f**) and quantification (**g**) of the intensity of phalloidin staining in axonal shafts in intact and transected explants. Scale bar: 50 μm. Data are represented as mean ± SEM.

NGF signaling is a positive regulator of growth cone morphology^16^ and hence GCC in degenerating axons early after TFW could simply reflect the sudden silencing of NGF signaling. We thus evaluated GCC in the distal portion of transected axons, a widely used model of nerve injury known as Wallerian degeneration (WD) that is executed in the presence of NGF. Axon transection was performed in DRG explants using a scalpel blade (Fig. 2a) and GCC assessed 3 hours later (before axon fragmentation, Fig. 2b,c). WD also induced GCC (Fig. 2d,e), but did not affect the global levels of phalloidin stained F-actin (Fig. 2f,g), in part because axonal tips whose growth cones have collapsed continue to have enrichment of F-actin staining (Fig. 2d, arrow heads). Taken together, these results show that GCC is an early event common to distinct modalities of axonal degeneration.

### Early Growth Cone Collapse After Degenerative Stimuli Can Be Dissociated From Long Term Axonal Fragmentation

**Figure 3:**
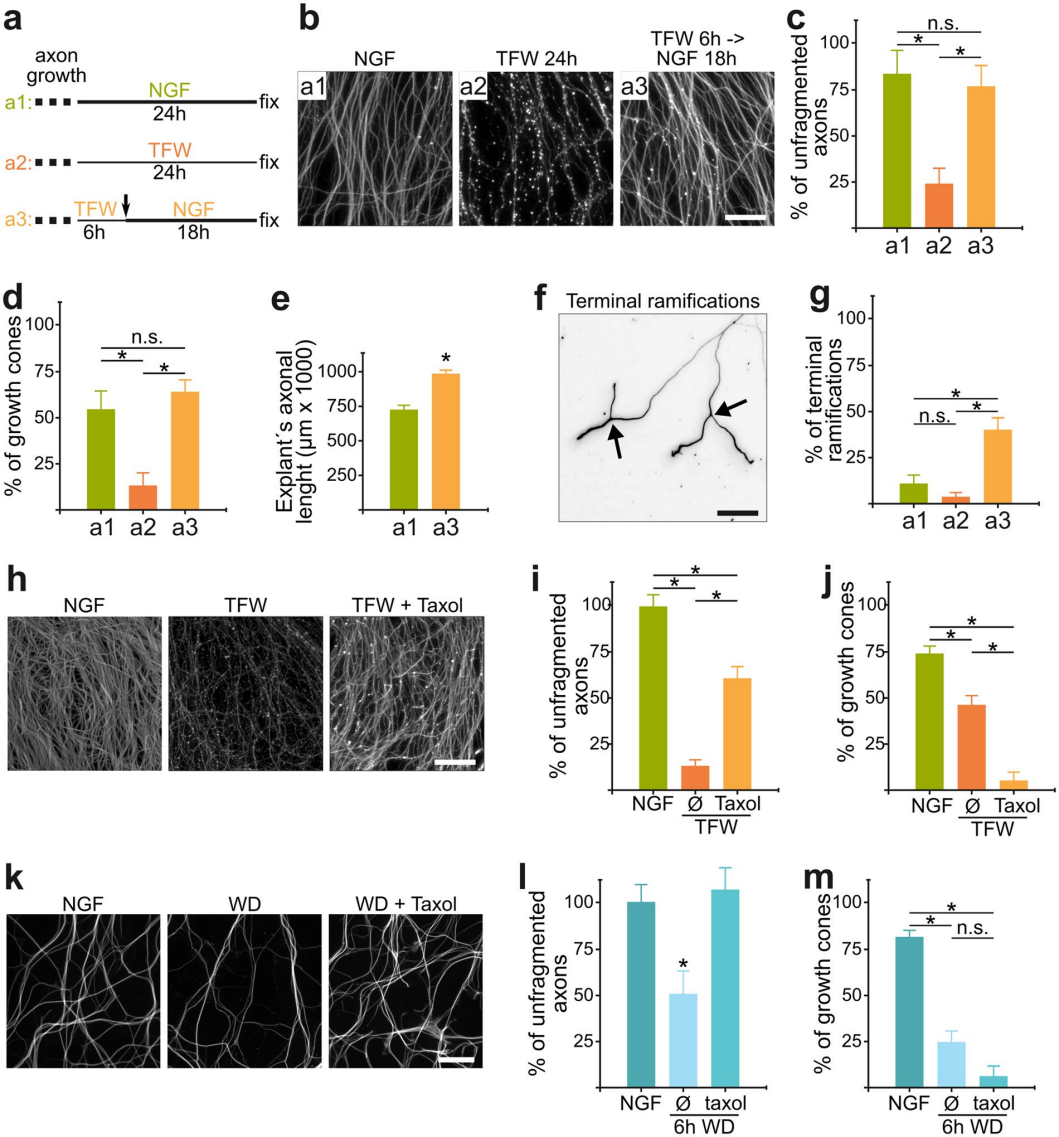
Early Growth Cone Collapse Can Be Dissociated From Long Term Axonal Fragmentation. (**a**) Scheme of treatments. After being in the presence of NGF for 3 days to allow axon growth, media is changed containing NGF (**a1**) or subjected to trophic factor withdrawal (**a2**), and fixed 24 hours later. To evaluate the reversibility of growth cone collapse, explants were subjected to TFW for 6 hours and then changed to a media with NGF and fixed 18 hours later (**a3**). Panels c, d, e and g use this letter-code for experimental treatments. (**b**,**c**) Representative images (**b**) and quantification (**c**) of the axonal area of control, deprived and NGF-rescued explants as specified in panel (**a**). Scale bar: 100 μm. (**d**) The proportion of growth cones in axonal tips is back to normal values in NGF-rescued axons. (**e**) NGF-rescued explants have larger axons than NGF-maintained explants. (**f**,**g**) Terminal branches (bifurcations close to the tip of axons, arrows in (**f**) are rare in control explants, but strikingly abundant in the rescued condition. Scale bar: 100 μm. (**h**,**i**) Representative images (**h**) and quantification of unfragmented axons (**i**) of control or 24-hours deprived explants in the absence or presence of taxol (10 μM). Scale bar: 100 μm. (**j**) Growth cone collapse induced by 3 hours of TFW is worsened in the presence of taxol (10 μM). Data are represented as mean ± SEM. (**k**,**l**) Axonal fragmentation induced by 6 hours of WD is significantly prevented by taxol 10 μM. Representative images (**k**) and quantification of unfragmented axons (**l**). Scale bar: 25 μm. (**m**) Growth cone collapse induced by 3 hours of WD is worsened in the presence taxol 10 μM. Data are represented as mean ± SEM.

We then examined the relationship between early GCC and late axonal fragmentation (the final step of axonal degeneration). We first asked if re-adding NGF can prevent axonal fragmentation after GGC is complete (6 hours, Fig. 3a), to determine whether GCC represents a point of no return towards axonal fragmentation. NGF is able to prevent axonal fragmentation when re-added up to 6 hours after deprivation (Fig. 3b,c). Interestingly, NGF-rescued axons re-form their growth cones (Fig. 3d), are longer (Fig. 3e) and more ramified towards their distal ends (Fig. 3f,g). Hence, GCC is reversible up to 6 hours after deprivation and does not represent a point of no return towards axonal fragmentation. The extended growth in the rescued explants might be related with deprivation increasing F-actin depolymerization (Fig. 1g,h), which after NGF re-addition will favor faster growth similar to the axon growth-promoting effect of acute treatment with the F-actin depolymerizing agent cytochalasin-D^17^.

To further investigate if GCC and fragmentation are mechanistically linked, we examined GCC in response to treatments that are known to prevent axonal fragmentation induced by either TFW or WD. As previously established^18^, treatment with the microtubule-stabilizing agent taxol (10 µM) added at the beginning of TFW prevent axonal fragmentation 24 hours later (Fig. 3h,i). However, taxol worsened GCC 3 hours after TFW (Fig. 3j). Similar results were obtained in the WD model (Fig. 3k–m). Together, these experiments show that loss of growth cones constitutes a robust degenerative event in itself, but may not be mechanistically related with later axonal fragmentation.

### Early and Sustained Remodeling of the Actin/Spectrin Membrane-associated Periodic Skeleton of Axons During Degeneration

**Figure 4:**
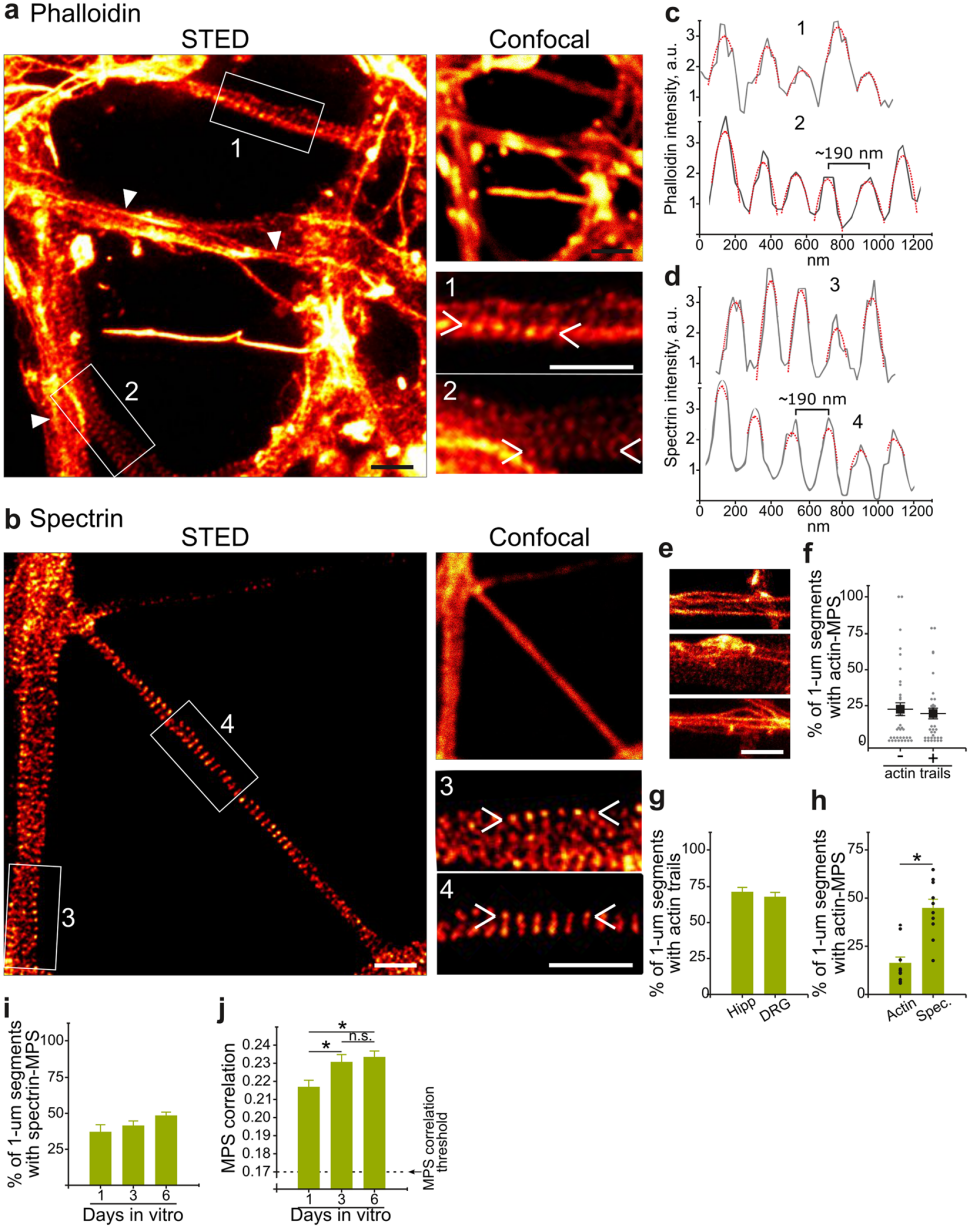
Actin/Spectrin-MPS in DRG explants. (**a**,**b**) STED images of phalloidin (**a**) and βII-spectrin (**b**) stained axons, including their confocal image (top right). Arrowheads in the inserts delimit the line used to trace the intensity profiles shown in (**c**) and (**d**) respectively. Scale bars: 10 μm (STED), 20 μm (confocal) and 1 μm (inserts). (**c**,**d**) Intensity profiles from inserts in panels a and b, evidencing the periodic distribution of phalloiding (**c**) and βII-spectrin (**d**) staining and its characteristic mean period of ~190 nm. (**e**) Representative images of axonal segments containing longitudinal actin fibers (actin trails). Scale bar: 1 μm. (**f**) The distribution of actin-MPS was independent from the presence of actin bundles in the same 1 μm-segment, since there is a similar abundance of actin-MPS in segments without actin bundles or segmente with actin bundles. (**g**) Abundance of actin trails were similar in axons from hippocampal primary neurons (7DIV) and DRG sensory neurons (3DIV). (**h**) Percentage of segments showing a periodic distribution of actin or βII-spectrin in DRG axons grown for 3 days *in vitro*. (**i**) Spectrin-MPS abundance of cultures at different days *in vitro*. (**j**) Correlation coefficients of segments with spectrin-MPS from cultures at different days *in vitro*. Data are represented as mean ± SEM.

We next examined other recently described F-actin structures throughout the axon during degeneration using Stimulated Emission Depletion (STED) nanoscopy. Super-resolution imaging of phalloidin- or βII-spectrin-stained DRG axons evidences the MPS (Fig. 4a,b) with the characteristic periodicity of ~190 nm (193 ± 21 nm for F-actin and 191 ± 28 nm for spectrin), as described^9,19^ (Fig. 4c,d). Because the MPS was suggested to progressively assemble from proximal to distal parts of the axon^11^, we always evaluated this structure at three quarters of the axonal length distally from the explant center. Also, this distance favored the evaluation of non-bundled or superimposed axons. Phalloidin staining also revealed longitudinal F-actin structures (Fig. 4a, arrowheads, and 4e), with no obvious spatial correlation with areas of MPS (Fig. 4f), suggesting that these two forms of F-actin arrangements are independent structures. These longitudinal bundles were recently described as a deep and dynamic F-actin pool referred to as “actin trails”^20^. Although the distinctive feature of actin trails is their active growth and shrinkage in living axons, they have also being described structurally using nanoscopy in fixed samples^20^ and hence we believe the longitudinal F-actin fibers we observe using STED nanoscopy most likely represent actin trails. We noticed a similar abundance of actin trails between axons from DRG and hippocampal neurons of similar length (Fig. 4g).

In order to quantitatively assess changes in MPS abundance and organization in an unbiased manner, we developed *Gollum*^21^, an open-source image analyses tool that automatically find axons, scans discrete segments and calculates their correlation with a user predefined modeled-MPS pattern (Supplementary Fig. S1 and S2, see materials and methods for details). We first determined the minimum correlation coefficient that corresponds to a visible periodical organization (*MPS correlation threshold*) in test images. Briefly, detected axons are scanned in sub-regions (1 µm × 1 µm) and the correlation coefficient of that segment is calculated (Supplementary Fig. S2). With the correlation coefficients of a group of images, we estimated two descriptive summary values, *MPS abundance* and *MPS correlation*. The *MPS abundance* refers to the proportion of sub-regions bearing correlation coefficients above the correlation threshold. And the *MPS correlation* is the mean correlation coefficient of segments considered to bear an MPS (i.e. that are above the correlation threshold), and evidences the regularity and level of organization of the periodical lattice (Supplementary Fig. S2).

We noticed that *Gollum* was more effective in βII-spectrin-stained images than in phalloidin ones. We believe this is due to a masking and confounding effect of the actin trails that are only visible in the latter. Also, as reported by others^11^, the visualization of the MPS by βII-spectrin was more clear and robust than by phalloidin staining, giving invariable superior *abundance* values when both stains were evaluated manually by an observer (Fig. 4h), likely due to the differential resistance of actin and spectrin structures to support the fixation procedure. On the other hand, actin trails were robustly evidenced across experiments. For these reasons, we primarily focused on spectrin staining analyzed with *Gollum* to assess changes in MPS during degeneration. Complementary, phalloidin STED images were quantified manually by a trained observer in blinded experiments. In these, the observer decides if a given sub-region presents at least 4 periods of ~190 nm, and MPS abundance is expressed as the proportion of axonal segments with such feature.

Because the MPS is formed after axon outgrowth and matures over time^11^, we first evaluated the maturation of the MPS of DRG axons as used in our experiments of trophic factor withdrawal. MPS abundance and correlation (i.e. organization) are at their maximum at 3 DIV, the time at which our experiments were performed (Fig. 4i,j). Interestingly, our unbiased and automated correlation analyses tool allowed us to observe a significant increase in organization of the MPS (increase correlation values) from 1 to 3 DIV (Fig. 4i), suggesting that once established, the MPS can further increase its organization over time.

**Figure 5:**
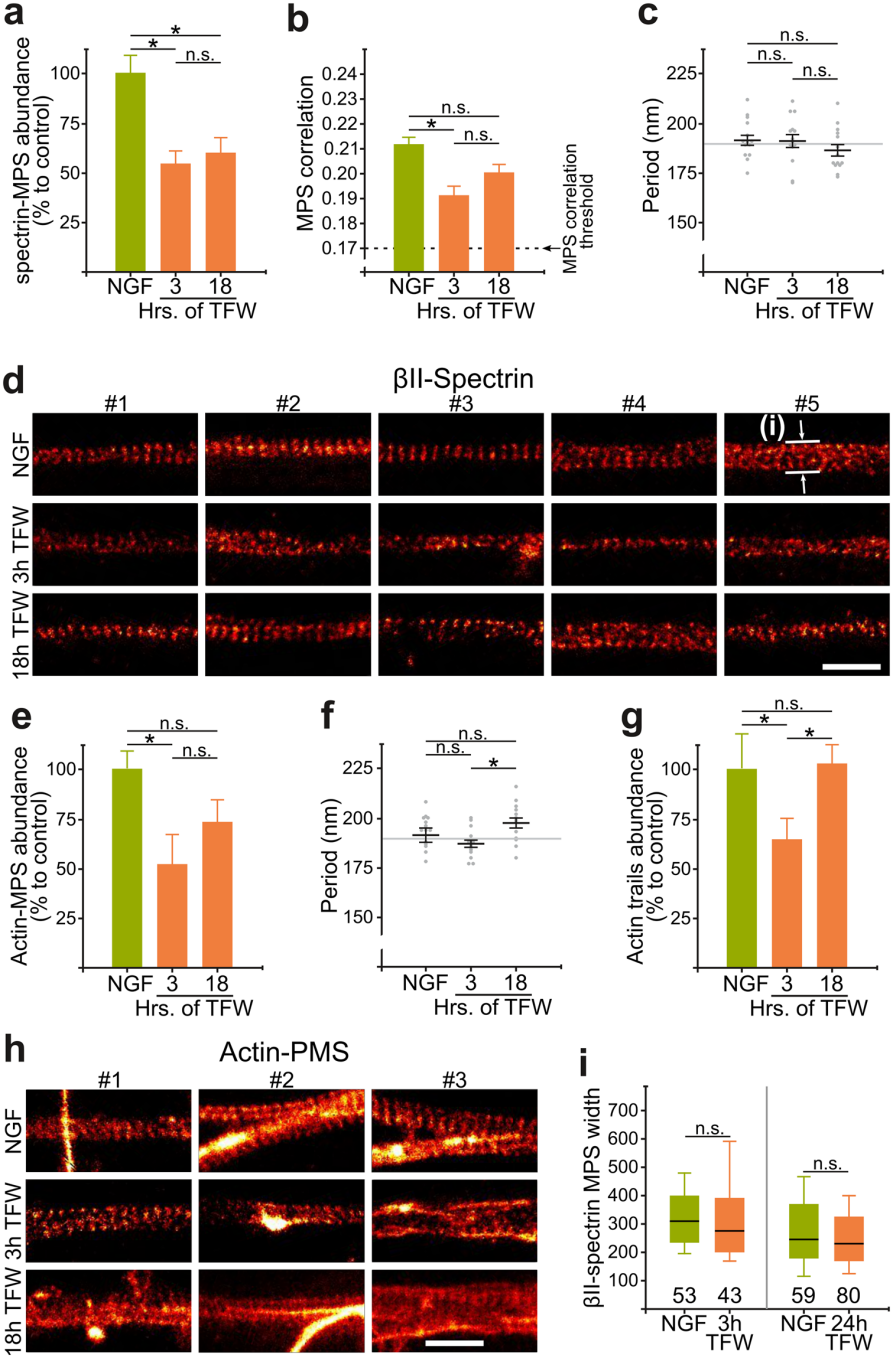
Remodeling of the MPS during TFW-induced degeneration. (**a**,**b**) βII-spectrin-MPS abundance (**a**) and correlation coefficients (**b**) after 3 or 18 hours of trophic factor withdrawal. (**c**) Mean period of βII-spectrin-MPS after trophic factor withdrawal. (**d**) Representative images of 5 segments with βII-spectrin-MPS in control explants or deprived for 3 or 18 hours. Scale bar: 1 μm. The top-right image shows an example of width measurements shown in panel (**i**). (**e**) Actin-MPS abundance after 3 or 18 hours of trophic factor withdrawal. **(f**) Mean period of actin-MPS after trophic factor withdrawal. (**g**) Abundance of longitudinal actin trails after trophic factor withdrawal. Data are represented as mean ± SEM. (**h**) Representative images of actin-MPS in control explants or deprived for 3 or 18 hours. 5 examples were chosen from each condition. Scale bar: 1 μm. (**i**) Box plots of βII-spectrin widths from STED images at different times after TFW. The solid box contains 50% of the data, while the bars include 95% of the data. The solid black line is the mean, and the numbers above the x-axis are the number of axonal segments measured in each condition.

We then quantitatively evaluated the MPS in axons undergoing degeneration. NGF deprivation induced a rapid ~50% drop in βII-spectrin- and actin-MPS abundance and correlation at 3 hours after TFW (Fig. 5a,e) and kept those levels at 18 hours after TFW. Eighteen hours is a time by which most axons start to show the different stages of the fragmentation process (see next sections). Axonal segments still displaying an MPS showed a drop in correlation levels (Fig. 5b), suggesting that during degeneration the MPS might exist in stages of stable intermediate organization. We did not observe significant changes in their periods (Fig. 5c,f), which is consistent with the proposed structural arrangement of the periodic lattice of short actin filaments spaced by spectrin tetramers^8^. When the MPS was evidenced with phalloidin and quantification made manually as described earlier we noticed a small recovery of MPS abundance at 18 hours after TFW. We believe this could be related to a slight gain in organization (but not abundance) of the MPS visualized by βII-spectrin staining towards 18 hours. Remarkably, the abundance of actin trails initially dropped (3 hours), but later recovered to control levels (18 hours, Fig. 5g). Figure 5d and h show examples of axonal segments displaying spectrin- (Fig. 5d) or actin-MPS (Fig. 5h) in control explants and after 3 or18 hours after TFW.

Adducin is part of a mature MPS^8,11^, likely binding to the barbed end of short F-actin filaments within the structure. Adducin null fly neurons show a 50% decrease in their MPS abundance^12^ and neurons from adducin null mice show an increase in MPS diameters^13^. This led us to evaluate if during degeneration there was an increase in MPS diameters, and therefore determined the width of βII-spectrin stained axons imaged by STED nanoscopy. We observed no changes in width as a function of degeneration (Fig. 5i).

### Late MPS loss is correlated with axonal fragmentation

**Figure 6:**
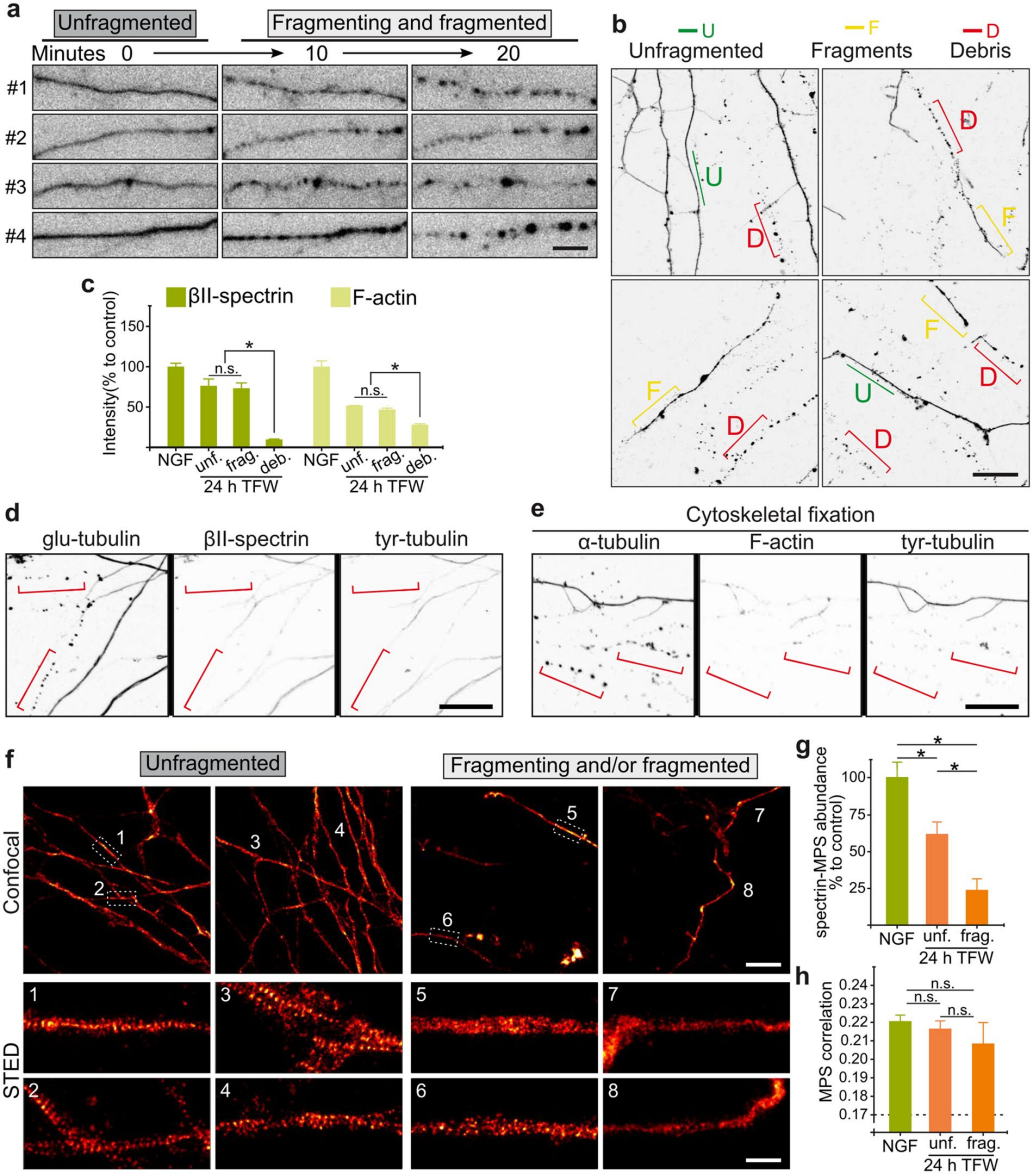
Dismantling of spectrin-MPS in fragmented axons. (**a**) Video-microscopy of axons expressing GFP and subjected to TFW for 24 hours. From 21 to 24 hours after TFW, axons suffer rapid fragmentation that is executed within ~20 minutes. (**b**) Explants fixed 24 hours after TFW and stained for microtubules would show degenerating axons at different stages of fragmentation (unfragmented, fragmented or debris). Scale bar: 20 µm. (**c**) Intensity of βII-spectrin and F-actin in axonal shafts from control axons, or axons deprived for 24 hours at different stages of fragmentation. (**d**,**e**) Representative confocal images stained for βII-spectrin (**d**) and F-actin (**e**) after 24 hours of TFW. Tubulin staining was used as a reference for the characterization of the different stages of fragmentation. Red brackets show debris, which is practically devoid βII-spectrin and F-actin. Scale bar: 20 µm. (**f**) Representative confocal (top) and STED (bottom) images of spectrin-MPS after 24 hours of trophic factor withdrawal in explants that are mostly unfragmented (left) compared to explants with a predominance of fragmented axons (right). Scale bars: 10 μm (confocal) and 1 μm (STED). (**g**,**h**) Spectrin-MPS abundance (**g**) and correlation coefficients (**h**) after 24 hours of trophic factor withdrawal in explants that are mostly unfragmented (unf.) compared to those that are fragmented (frag.).

**Figure 7:**
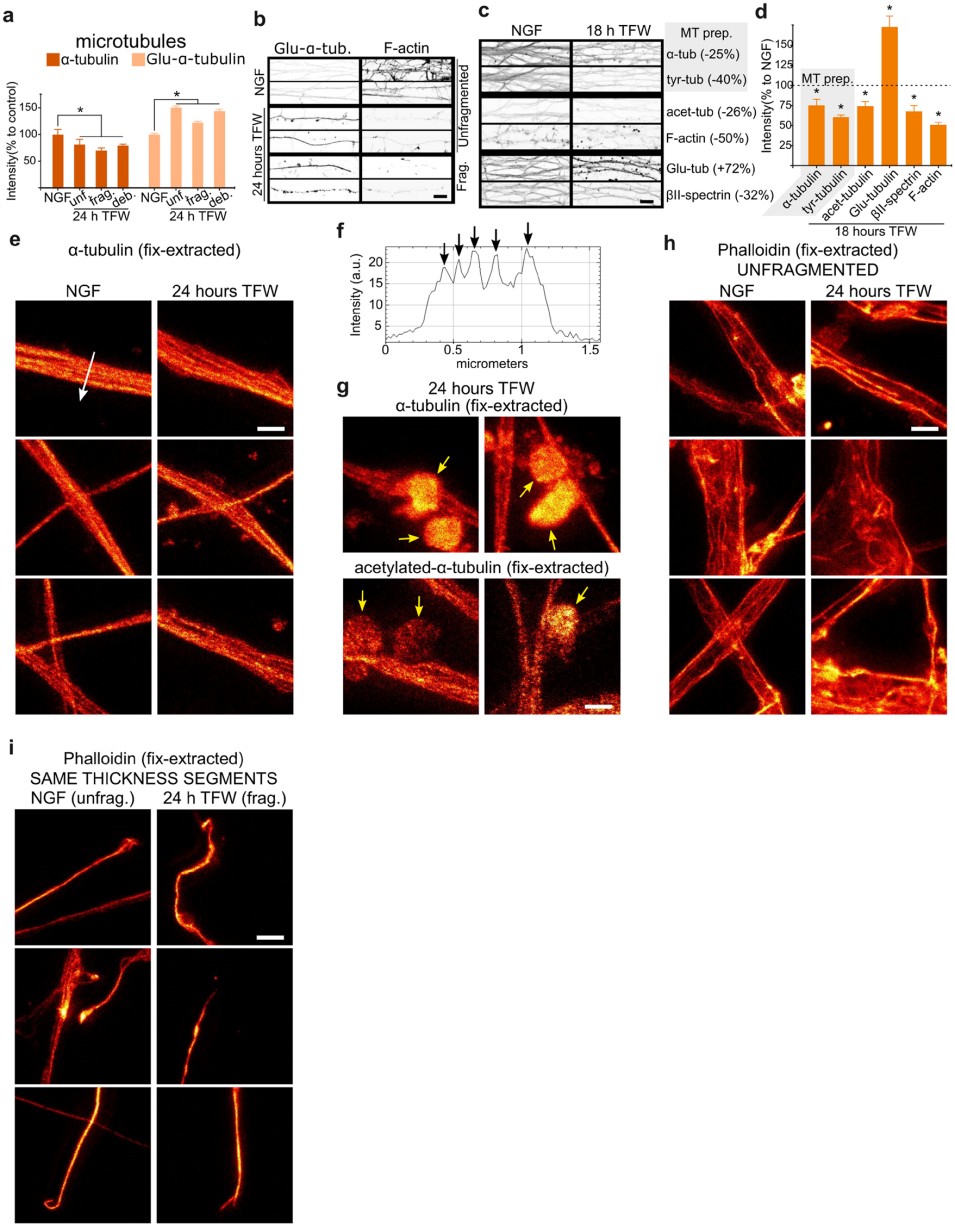
Microtubules and actin trails during fragmentation. (**a**) Intensity of microtubular α-tubulin and glu-α-tubulin in axonal shafts from control axons, or axons deprived for 24 hours at different stages of fragmentation. (**b**) Representative quantitative confocal images of glu-α-tubulin and F-actin (for reference) of control axons, or axons deprived for 24 hours which are unfragmented or fragmented. Scale bar: 10 μm. (**c**,**d**) Representative quantitative confocal images (**c**) and quantification (**d**) of tubulin variants, βII-spectrin and F-actin of control axons, or axons deprived for 18 hours. Microtubule preparations (MT prep.) were achieved by detergent-extraction during glutaraldehyde fixation. Values between brackets in (**c**) are the mean change compared to the NGF control condition. Scale bar: 10 μm. Data are represented as mean ± SEM. (**e**) Representative axonal segments with microtubules stained against α-tubulin and images with STED nanoscopy. Scale bar 1 µm. (**f**) intensity profile from the line showed in panel (**e**). Arrows point to intensity peaks evidencing individual microtubules along the axon. (**g**) Representative STED images from microtubules stained against total α-tubulin (top) or acetylated-α-tubulin (bottom). Arrows point to debris where microtubule staining does not evidence filamentous polymers. Scale bar 1 µm. (**h**,**i**) Representative STED images of phalloidin staining on fixation-extracted axons. Scale bar 1 µm.

Axonal degeneration culminates with the fragmentation of the axon. We then asked whether the MPS is maintained during fragmentation or if it would persist in the axonal fragments. In order to recognize the different stages of axonal fragmentation, we used time-lapse video microscopy of axons expressing soluble GFP. By 18–24 hours of TFW, axons start to form consecutive bead-like structures that grow in size until they lose connection with each other (Fig. 6a), effectively breaking the axon into small round structures that we will refer as *debris*. Because along an individual axon this process may happen at slightly different times, linear axon fragments are transiently produced, that we will refer as *fragmented*. The same fragmentation features can be observed in fixed samples 24 hours after TFW and stained for microtubules (Fig. 6b), allowing us to compare directly the MPS during the different stages of fragmentation. βII-spectrin and F-actin levels (as assessed by semi-quantitative confocal microscopy) show a significant decrease by 24 hours of TFW, especially in F-actin, but there is no difference between unfragmented or fragmented axons (Fig. 6c). On the other hand, βII-spectrin and F-actin are almost completely lost in axonal debris (Fig. 6c–e). Interestingly, STED images of βII-spectrin staining show that the MPS is mostly absent from fragmented axons (Fig. 6f–h). Since the intensity of βII-spectrin and F-actin was similar in both populations of axons (unfragmented and fragmented.) (Fig. 6e), the sharp loss of MPS is not due to the mere loss of its constituents but rather to a specific dismantling of MPS organization. We then asked if MPS disassembly during fragmentation represents a widespread loss of cytoskeleton components and examined changes in microtubules and actin trails during degeneration. We found that microtubules, as revealed by staining of cytoskeletal preparations (mild lipid extraction during fixation under microtubule-stabilizing conditions^22^) with an antibody against α-tubulin, slightly decrease in deprived axons, but no further drop was detected during fragmentation (i.e. in fragmented axonal segments or debris, Fig. 7a). We also found an unexpected increase in detyrosinated-α-tubulin (*glu*-microtubules) staining (Fig. 7a,b), suggesting that degenerating axons might be enriched in stable polymer^23^. Because taxol treatment suggests that microtubule stabilization might prevent axon fragmentation^18^, we further explored changes in microtubules in degenerating axons before fragmentation, comparing the levels of tyrosinated-, detyrosinated- and acetylated α-tubulin populations in microtubules after 18 hours of TFW. In contrast to a previous report^18^, we consistently observed an increase in detyrosinated α-tubulin (glu-microtubules) staining during degeneration, with a concomitant decrease in tyrosinated-α-tubulin (dynamic microtubules) staining of membrane-extracted axons (Fig. 7c,d). It is well established that tubulin acetylation occurs on glu-microtubules^23^; however, and despite the increase in glu-microtubules staining, a minor decrease of acetylated α-tubulin staining was detected after TFW. STED microscopy of detergent-extracted axons stained for α-tubulin revealed that microtubules in control and deprived axons (unfragmented or fragmented) appear similar, when comparing axons of the same width (Fig. 7e). In axons it is difficult to identify individual microtubules, because of their compact bundling^24^, but our STED imaging could still depict distinct microtubule “tracks” (Fig. 7f, arrows). The round bead-like structures that we refer to as “debris” were also positive for microtubules, since they were stained for α-tubulin in detergent-extracted preparations and for acetylated-microtubules (Fig. 7g), however the staining of these structures did not allow distinguishing individual tracks. We also evaluated if actin trails would persist at late time points of degeneration, by performing STED nanoscopy on phalloidin-stained lipid-extracted axons. The abundance of longitudinal F-actin structures was similar between unfragmented axonal segments from control or 24 hours after TFW, although in the latter, actin trails were smaller in length (Fig. 7h). On the other hand, F-actin structures found in fragmented (deprived) axons were similar to those observed in control axonal segments of the same width (Fig. 7i). These results show that the loss of the MPS occurs during fragmentation and that it precedes the loss of microtubules and actin trails, since these last two cytoskeletal elements are still present in fragmented axons.

**Figure 8:**
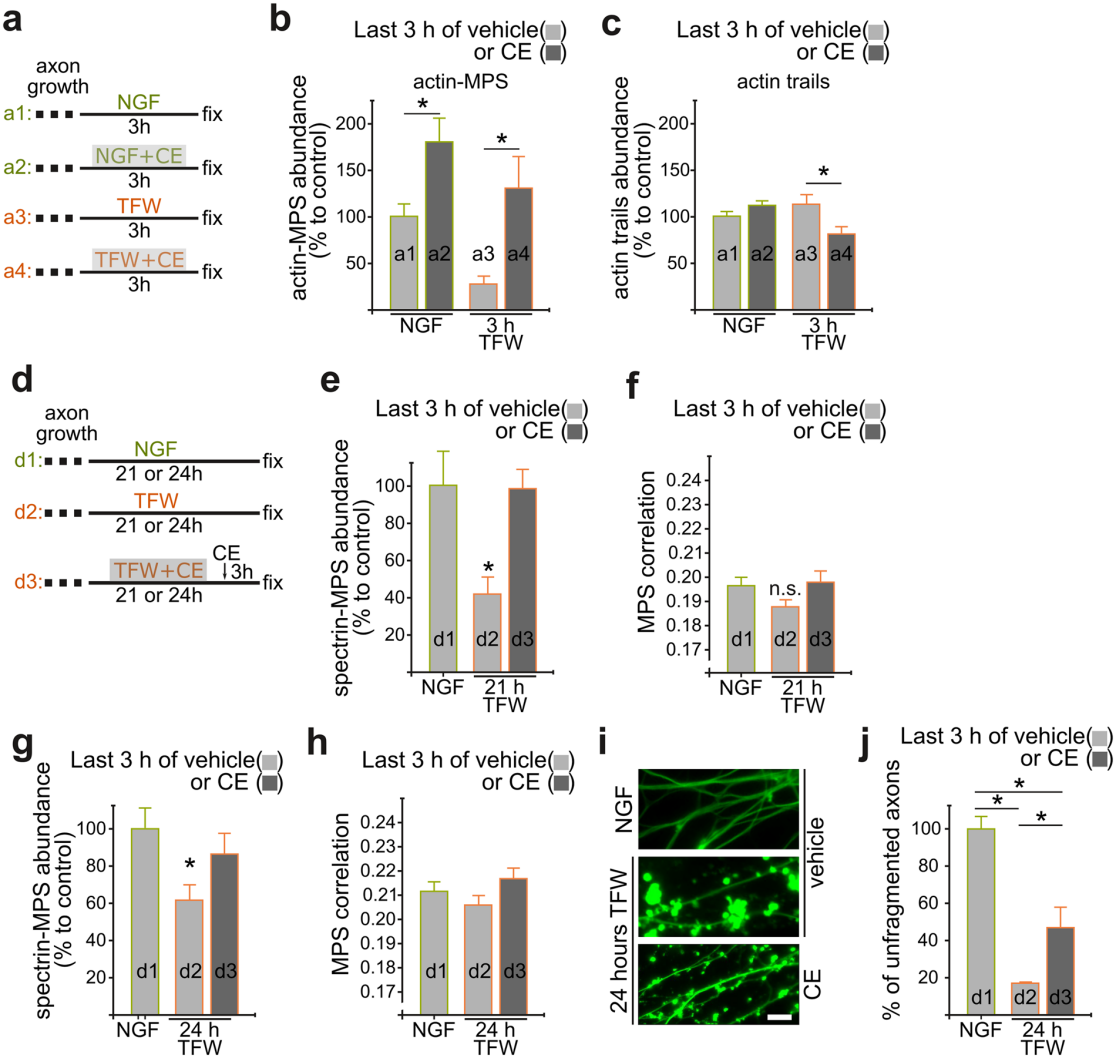
Cucurbitacin E prevents MPS remodeling and ameliorates axon fragmentation. (**a**) Scheme of treatments. After being in the presence of NGF for 3 days to allow axon growth, media is changed containing NGF (a1 and a2) or subjected to trophic factor withdrawal (a3 and a4), and fixed 3 hours later. Some explants received cucurbitacin E (5 nM, a2 and a4) for the 3 hours. (**b**) With the design shown in (**a**), CE was able to increase the abundance of actin-MPS in control conditions and after TFW. (**c**) Acute treatment with CE did not increase the abundance of actin trails. (**d**) Scheme of treatments. After being in the presence of NGF for 3 days to allow axon growth, media is changed containing NGF (d1) or subjected to trophic factor withdrawal (d2 and d3), and 21 or 24 hours later some received CE for 3 hours (d3). (**e**–**h**) CE added 3 hours before fixation at 21 (**e**,**f**) or 24 (**g**,**h**) hours after TFW revert spectrin-MPS abundance and correlation coefficients close to NGF control values. (**i**,**j**) Representative images (**i**) and quantification (**j**) of axons of control explants and explants subjected to 24 hours of TFW in the absence or presence of CE in the last 3 hours before fixation. Scale bar: 10 μm. Data are represented as mean ± SEM.

In order to determine if MPS stability is necessary for axonal fragmentation, we evaluated if MPS maintenance would ameliorate axon fragmentation in deprived axons. We found that low concentrations the F-actin stabilizing drug cucurbitacin E^25^ (CE, 5 nM) increases the abundance of MPS after acute 3-hour treatments of either NGF or NGF-deprived axons (Fig. 8a,b), but does not increase actin trails abundance (Fig. 8c). Of all cucurbitacins, CE is the most specific for modifying free cysteines in F-actin. Also, the low doses used here are below the concentrations that were characterized for off targets^25^. Also, CE added 3 hours before fixation in explants deprived for 21 or 24 hours reverted MPS abundance and organization to control levels (Fig. 8d–h). More importantly, this late treatment significantly ameliorated axonal fragmentation (Fig. 8i,j), suggesting that during degeneration the MPS is dismantled before axonal fragmentation and that MPS loss is necessary for fragmentation.

## Discussion

**Figure 9:**
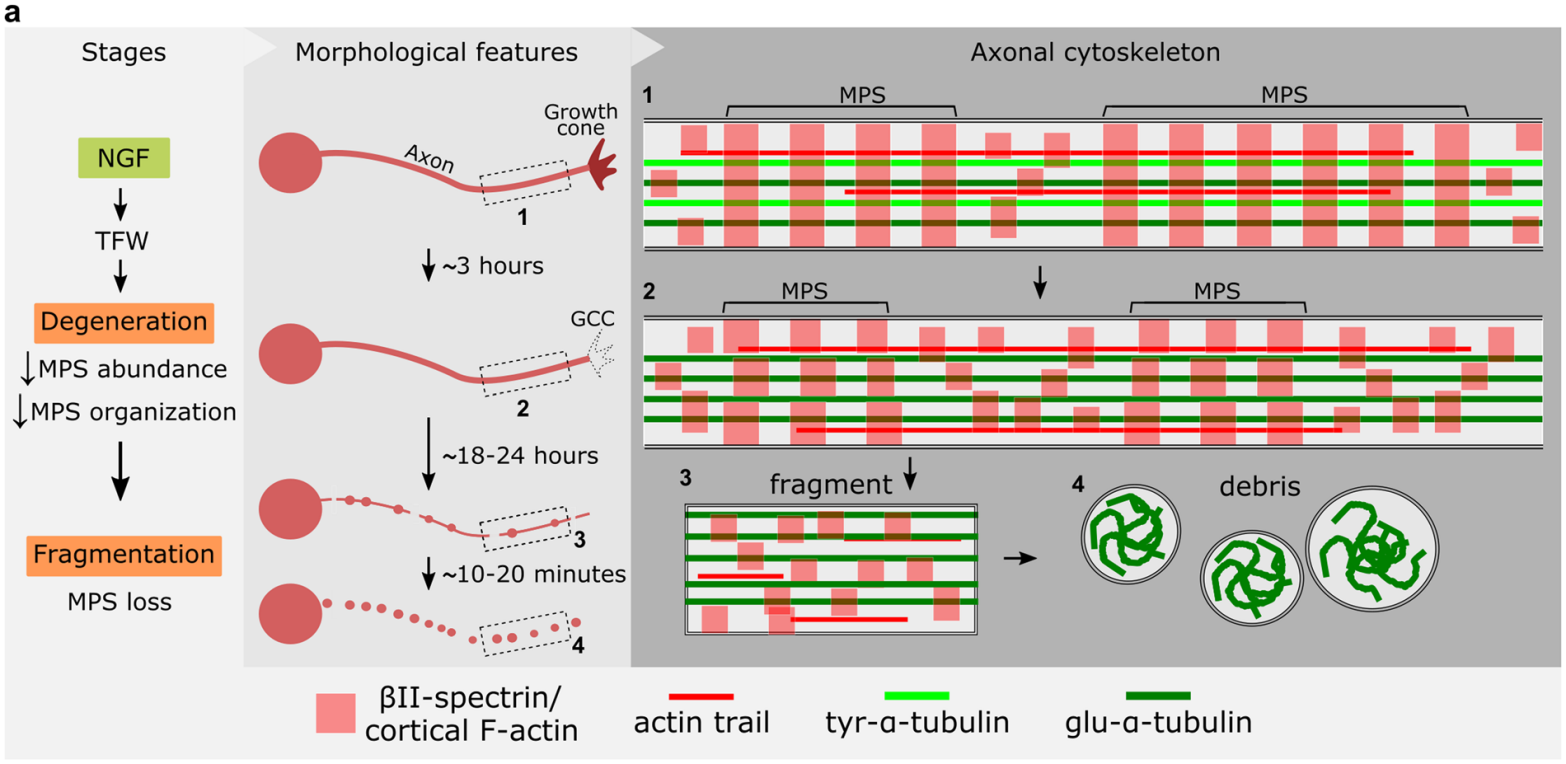
The axonal cytoskeleton during developmental pruning. For most of the degeneration period (3–18 hours after TFW) growth cones collapse, and the MPS loses organization and its abundance decreases by ~50%. At the same time, microtubules get enriched in detyrosinated tubulin. During fragmentation, the MPS is lost, but βII-spectrin and F-actin are still present. When fragments become round beads-like structures, βII-spectrin and F-actin are almost absent, while they continue to be positive to markers of microtubules.

Using a physiologically-inspired model, we studied the reaction of various actin cytoskeletal structures along the axon during degeneration. We found early and complete GCC, accompanied by a global decrease in F-actin and a remodeling of the MPS that lasted until fragmentation. We found that axons in the process of fragmentation lose their MPS, but not their microtubules, and pharmacologically maintaining the MPS significantly ameliorated axonal fragmentation (Fig. 9).

Unbiased proteomics of injured axons have repeatedly shown an enrichment of actin cytoskeleton regulators^6,26,27^. Actin regulators have also been linked to axon maintenance and axonal degeneration in development and in neurodegenerative conditions^2,3,5,28^. These reports suggested that the actin cytoskeleton is altered in degenerating axons and that it is a necessary step in axon degeneration. Our finding of the fast and sustained reaction of F-actin-rich structures to TFW indicates that the early and sustained remodeling of the actin cytoskeleton is a major component of the morphological changes axons exhibit during degeneration.

GCC allows the growing axon to re-direct its trajectory away from inhibitory cues^14^. In the context of the so called “neurotrophin hypothesis”^29^ during development of the sensory system, GCC might provide “loosing” axons with the opportunity to re-direct their growth towards a pro-survival source. In this regard, our NGF-rescue experiments highlight the ability of the axon stump to regenerate a growth cone providing the proper trophic support conditions are timely met. On the other hand, the presence of GCC in distinct forms of degeneration (TFW and WD) raises the possibility of using GCC as an early morphological marker of axonal degeneration.

Nanoscopy has recently unveiled F-actin structures within the axon: actin trails consisting of deep, longitudinal F-actin bundles^20^, and the MPS^8^. Actin trails have been proposed to function as carriers of short actin filaments. We found that the abundance of actin trails during degeneration initially decreases, and then recovers to normal levels, suggesting that these structures do not contribute directly to late axonal fragmentation.

The finding of MPS in axons from worms to humans supports the speculation that this supra-molecular arrangement might be essential for the physical integrity of axons. In favor of this, motor axons from *C. elegans* spectrin nulls break as the nematode swims^30^. However, there were no evidence of MPS remodeling in a physiological condition compromising axon integrity. Here, we show a correlation between MPS and axon maintenance in a physiological condition.

Our results show that the MPS reacts to degeneration in a two-tier way: *before* and *during* axon fragmentation. Initially, TFW induces a rapid and long lasting 50% decrease of MPS abundance that lasts until fragmentation of the axon, that we believe represent a different steady-state where the rates of MPS assembly and disassembly are altered and balance at a different equilibrium point. Treatments with small molecules known to alter the polymerization and depolymerization of actin filaments support the notion that the MPS is not a fixed structure but rather exist, not surprisingly, in a *dynamic equilibrium*. Unlike jasplakinolide, which stabilizes F-actin but also binds to G-actin, cucurbitacin E interacts with and stabilizes only F-actin^25^. The fact that a 3-hour treatment with CE is sufficient to increase MPS abundance is suggestive that MPS components are not fixed in time but are rather exposed to a relatively fast turnover. This dynamic equilibrium model is also supported by previous reports. For example, a 1-hour treatment with drugs that prevent actin polymerization (latrunculin A or cytochalasin-D) are sufficient to completely dismantle the MPS from hippocampal or fly axons, suggesting that F-actin within the MPS is constantly exchanging G-actin^11,12^. Moreover, genetically manipulating the levels of spectrin correspondingly affect MPS abundance^11,12^.

Axonal fragmentation represents the end point of the degeneration process. Our data suggests a crucial role for MPS at this stage, whereby this structure is to be removed in order for fragmentation to proceed at a normal rate: fragmenting axons lack MPS and acutely preventing MPS disassembly at late time points ameliorates axon fragmentation. MPS loss during fragmentation is rather unique for this cytoskeletal component, since microtubules persist in axonal fragments and even in axonal debris. Our data further suggests that these microtubules are enriched in stable polymer, since they contain detyrosinated tubulin at the expense of the tyrosinated form. These observations are at odds with a previous report showing that all forms of tubulin were significantly decreased in degenerating explants^18^. Differences in sample processing and analysis may account for the diverse observations: while we evaluate tubulin forms in extracted axons (evidencing only tubulin forms within microtubules), the previous study examined total homogenates (monomeric tubulin plus polymeric one) from whole explants (cell bodies and axons). Regardless of this discrepancy, previous evidence and data presented here support the notion that microtubules and the MPS are physically and functionally related. Microtubules and the MPS can interact through ankyrins, which can bind simultaneously to both cytoskeletal structures and have been shown to be distributed periodically in axons^8,31^. Moreover, microtubule disassembly with nocodazole destroys the MPS^11^. Conversely, fly neurons treated with cytochalasin-D affects microtubule dynamics, a phenotype that is worsened with the concomitant genetic deletion of the microtubule-stabilizing protein Shot or treatment with nocodazole^12^ and ultimately leads to axon loss. In axons undergoing degeneration in a physiologically-inspired condition, we observe that the MPS is lost right before fragmentation, whereby microtubule tracks are still present. Since taxol is able to protect these axons from fragmentation, it was puzzling to observe that normal axon fragmentation take place in the presence of microtubules. One possibility is that taxol is actually having a protective effect by preventing other aspects of microtubule functioning that go beyond solely maintaining polymer mass. For example, taxol could be preventing a key dynamic behavior of microtubules, the proper movement of motor proteins and cargoes or could be affecting the association/dissociation of relevant microtubule-associated proteins. For example, it was recently reported that TFW requires signaling between the axon and the soma to initiate nuclear transcription whose product have to travel back to the axon to trigger degeneration^32^. Hence, it is possible that taxol, by affecting microtubule-mediated transport^33,34^, impairs this signaling and hence axonal degeneration. Dissecting the exact molecular mechanism by which taxol prevents axonal degeneration, other than maintaining microtubule mass, require further study.

So far, quantitative analysis of the MPS has been performed by autocorrelation analysis with manual selection of regions-of-interest aiming to determine the “degree of spectrin/actin periodicity”^11^ or having a trained observer decide over the presence or not of the MPS^12^. However, this approach has severe limitations. First, the regions of interest are handpicked with the risk of introducing subjective bias. Secondly, because such a task is highly time-consuming, only a relatively small number of neurons can be analyzed, limiting the statistical relevance of results. Quantitative studies of the MPS call for specific and automated image analysis tools that overcome these two major drawbacks, enabling reproducibility as well as the identification of subtle, but probably physiologically relevant changes. For this, we have developed a method and its implementation as an open-source image analysis tool for the automated quantification of the abundance and quality of protein periodic structures in images of biological samples, dubbed *Gollum*^21^. The application can be downloaded from a public repository (see Methods) and allows for the systematic and quantitative assessment of the MPS involving larger numbers of images and all possible sub-region in each image. Its application in the current study allowed us to identify not only changes in abundance but also in the more subtle changes in organization of the periodical lattice. We believe that automated image analyses tools like Gollum will significantly help to advance our understanding of the biology of the MPS.

In summary, our observations unveil significant remodeling of the actin cytoskeleton in critical axonal domains and at critical phases of the degeneration process and suggests a key role of the MPS in maintaining axon integrity during degeneration.

## Methods

### Antibodies and Reagents

Antibodies directed against βIII tubulin (Tuj-1, 1:1,000 for immunofluorescence) were obtained from Millipore. Cucurbitacin-E (5 nM, SML0577), Paclitaxel (Taxol, 10 µM), mouse monoclonal anti-acetylated tubulin (Clone 6-11B-1) and anti-alpha-tubulin (clone DM1A) were from Sigma. Phalloidin-ATTO647N (used for STED) was from ATTO-TEC and phalloidin-rhodamine (used for widefield) from Thermo Fisher. Rat anti-tyr-tubulin antibody was from Abcam (clone YL1/2, cat#ab6160). Rabbit anti-glu-tubulin was a kind gift from Dr Gregg G. Gundersen (Columbia University Medical Center, US) and was used previously^35^. Mouse anti-βII-spectrin was from BD Biosciences (US, cat#612563).

### DRG Culture and Treatments

C57Bl/6 mice were provided by our Specific Pathogen Free Animal Facility. All procedures involving animals were approved by the institution´s (INIMEC-CONICET) Council of Animal Care, by National Department of Animal Care and Health (SENASA, Argentina) and were in compliance with the general guidelines of the National Institute of Health (NIH, USA). Efforts were made to minimize the number of manipulations and the animals used. DRG explants were prepared from E13.5 mouse embryos and grown on cell culture 18 mm coverslips coated sequentially with poly-D-lysine (1 mg/ml; Sigma-Aldrich), laminin (10 mg/ml; Sigma-Aldrich), and collagen (0.1 mg/ml, PureCol; Advance BioMatrix). The basal culture media consisted of Neurobasal (Invitrogen) supplemented with 2% B-27 (Invitrogen), 1% L-glutamine (Wisent), 1% penicillin/streptomycin (Wisent), and 20 mM 5-fluoro-20-deoxyuridine (Sigma-Aldrich).Axon extension was performed in the presence of Nerve Growth Factor (NGF, Alomone Labs). NGF deprivation was achieved by changing the media to an NGF-free basal medium supplemented with anti-NGF antibody Rab1 (1 mg/ml^36^). Axotomy was achieved *in vitro* by transecting one side of the explant and putting them into the incubator for different times as indicated in the text. 1.

### Hippocampal cultures

Cultures of hippocampal neurons were prepared from Wistar rats at embryonic day E18. Tissue dissociation was performed by incubating in 0.25% Trypsin for 15 minutes at 37 °C, and mechanically by passing through fire-polished glass Pasteur pipettes of increasing gauge. Cells were plated on coverslips coated with 1 mg/ml poly-L-lysine (Sigma-Aldrich). Neuronal cultures were maintained in Neurobasal medium (Gibco), supplemented with 2% B27 serum-free supplement (Gibco), Glutamax (Gibco, cat) and pen/strep. Medium was replaced once per week.

### Immunocytochemistry and microtubule preparations

DRG explants were fixed with 4% paraformaldehyde for 10 min at room temperature and then blocked in blocking solution containing 5% BSA, and 0.3% Triton X-100 in PBS. The DRGs were then incubated with primary antibodies for 1 hour at room temperature and secondary antibodies for 1 hour at room temperature. Coverslips were then mounted in slides with a home-made mounting media based in Mowiol (2.4% Mowiol 4–88 (poly(vinyl alcohol), Sigma) and the anti-fade reagent DABCO (2.5% w/v, 1,4-diazobicyclo[2.2.2]octane, Sigma), as described previously^37^. The secondary antibody used for STED was an ATTO-647N goat anti-mouse. When indicated, detergent extraction was performed during fixation to “wash” the preparation from soluble cytosolic components of the axons using a “microtubules preparation” protocol previously described^22^. Briefly, explants were extracted/fixed at RT for 20 minutes using the following solution: 60 mM PIPES, 25 mM HEPES, 5 mM EGTA, 1 mM MgCl (pH 6.9), 0.25% glutaraldehyde, 3.7% paraformaldehyde, 3.7% sucrose and 0.1% Triton X-100.

### Quantification of Axonal Degeneration

Axonal degeneration quantification was performed essentially as described previously^38^. In brief, blinded anti-tubulin-stained samples were imaged with a systematic random sampling approach, with 3–4 images per explant, 400 µm away from the center of the explant, harvesting at least 20 images per treatment. Using the NIH ImageJ software, the axonal area was determined by the total number of detected pixels after binarization of the image using the threshold function. To estimate the area occupied by the pixels from “unfragmented axons, fragmented axons and debris were excluded by size (defined as small particles using the ImageJ particle analyzer function). At least two coverslips were evaluated for each condition and each experiment was repeated three times.

### Quantification of growth cone collapse

Explants were double stained against α-tubulin and F-actin (phalloidin). Under the microscope, a trained observer blind to treatment assessed the proportion of axonal tips with a growth cone for each randomly-visited field of view. Growth cones were defined as F-actin enrichments in the tips of tubulin-stained axons that either show an expanded lamella and/or 3 or more filopodia protrusions. Percentage of growth cones was determined as the proportion of axonal tips with a growth cone multiplied by 100.

### Video microscopy

Time lapse microscopy was performed using DIC filters in a Zeiss inverted microscope, at a rate of one image every 3 minutes to evaluate growth cone morphology with a 60X oil-immersion objective (Figure S1). Also, time lapse microscopy was performed in DRG explants derived from Isl-1 cre: ROSA-tau-GFP, were the tau-GFP reported was expressed in axons of DRG neurons and detected using a LED inverted fluorescent microscope (Zeiss, Figure S6), at a rate of 1 picture every 5 minutes.

### Stimulated Emission Depletion Nanoscopy

The STED nanoscope was home-built (see Supplementary Fig. S3 for a comprehensive scheme of the nanoscope). For excitation, the setup is prepared to use two colors, although in the present work only one was used (640 nm): (i) 592 nm light was selected with an interference filter (Chroma ZET594/10X) from a pulsed (430 ps) super-continuum laser (Fianium FemtoPower1060) operating at 40 MHz repetition. It was linearly polarized using a polarization beam splitter. (ii) A linearly polarized pulsed (200 ps) laser at 640 nm (PicoQuant LDH-P-C-640B) operating at 20 MHz repetition. Each color was coupled into a polarization maintaining single-mode fiber (PMF1 and PMF2; Thorlabs P3-488PM-FC-5) using a fiber collimator (L1 and L2; Schäffer und Kirchoff 60FC-4-A7.5-01). Light exiting the fiber was collimated (L4 and L5; f = 30 mm) in order to obtain the TE00 excitation beams and circular polarization was adjusted for each color using a broadband (400 nm – 800 nm) quarter-wave plate (λ/4; ThorlabsAQWP05M-600) and a broadband (400 nm – 800 nm) half-wave plate (λ/2; ThorlabsAHWP05M-600).

For STED, a linearly polarized pulsed (1 ns) laser at 775 nm was used (Onefive Katana HP). Light was coupled into a polarization maintaining single-mode fiber (PMF3;Thorlabs P3-630PM-FC-5) using a fiber collimator (L3; Schäffer und Kirchoff 60FC-4-A11-02). STED light coming out of the fiber was then collimated (L6; f = 30 mm) and sent through a 0 − 2π phase plate (VPP; RPC Photonics VPP-1a). Circular polarization was adjusted using a broadband (690 nm–1200 nm) quarter-wave plate (λ/4; Thorlabs AQWP05M-980) and a broadband (690 nm–1200 nm) half-wave plate (λ/2; ThorlabsAHWP05M-980).

Excitation and STED beams were combined using a long-pass dichroic mirror (D1; Chroma ZT594RDC), notch filter at 22 degrees (NFE; SemrockNF03-658-25) and 5 mm short-pass dichroic mirror (D2; Chroma Z780sprdc) respectively. Lateral beam scanning was performed by a system composed of a home-made galvanometric scanner, a scanning lens (SL; Leica) and a tube lens (TL; Leica). For additional positional control and fine focusing, the sample was mounted on a XYZ piezo electronic nano positioning stage (Thorlabs NanoMax MAX311D/M). Light was focused to the diffraction limit with an objective of 1.4 NA (O; Leica HCX PL APO 100x/1.40-0.70 Oil CS).

Proper alignment of the three beams was performed measuring scattering of 40 nm gold nanoparticles. A magnetically mounted pellicle beam splitter coated for 400 nm–700 nm, 45:55 (R:T)(mP; Thorlabs BP145B1) directed scattering light to a broadband (280 nm–850 nm) photomultiplier tube (PMT; Thorlabs PMM02).

STED wavelength was rejected from the detection path using a notch filter (NFF; SemrockNF03-785-25), fluorescence was divided into two channels using a long-pass dichroic mirror (DF; Chroma ZT647RDC) and light for each channel was selected using band-pass filters (FF1; Semrock FF01-623/24-25 and FF2;Semrock FF01-676/37-25). Light was then coupled (L8 and L9; f = 50 mm) into a 25 µm, 0.1 NA multi-mode fiber (MMF1 and MMF2; Thorlabs M67L01) of the confocal detector (APD1 and AP2; MPD PD-050-CTC-FC). Time gating of fluorescence photons was performed using a custom-made electronic board (MPI for biophysical Chemistry). A schematic representation of the STED nanoscope is shown below.

### Manual assessment of MPS abundance

STED images (10 µm × 10 µm) were divided in 1 µm × 1 µm sub-areas and a trained observer blind to treatment decided visually whether the segment presented or not periodicity in the staining (see Materials and Methods for more details). MPS abundance was then calculated as the percentage of segments with an MPS over the total number of segments with axonal material.

### Assessment of MPS abundance and organization using the image analysis tool GOLLUM

In order to asses quantitatively MPS abundance and organization in an unbiased manner, we develop an image analyses tool that we named *GOLLUM* and a detailed description of its working principle and capabilities was recently published^21^. Briefly, the algorithm scans the image in 1 µm × 1 µm squares and calculates its correlation with a MPS modeled with Gaussian peaks 190 nm apart. The correlation value is indicative of how preserved is the MPS in that ~1 um stretch, allowing for the detection of mild changes in MPS organization. The algorithm is effective in both phalloidin- and βII-spectrin-stained axons, but is more accurate in βII-Spectrin stained axons and we thus used Gollum for these. We first determined the correlation that corresponded to a visible MPS, 0.17, and then used it for all experiments. MPS abundance refers to the proportion of squares with correlation values above the defined threshold and the MPS correlation is the mean correlation of those squares above the threshold (with MPS). The *GOLLUM* software set is entirely written in Python 3 with Qt as the graphical user interface (GUI) framework, making it cross-platform. The source code, Python executable programs with graphical user interfaces, and detailed documentation are maintained at a public repository in https://github.com/cibion-conicet/Gollum.

### Periods assessment

For the quantification of the mean period of phalloidin and βII-spectrin stainings, a line profile (3 pixels averaged) was laid across 4–5 consecutive ring-like structures on the images. The peak of each periodic structure was found by fitting a quadratic function to points around local maxima of the profiles. The mean distance between two peaks was used as a characteristic period of that particular segment. This analysis was performed with custom written Matlab routines. For each experimental condition, we collected at least 15 of these mean periods to estimate the period of the treatment.

### Statistical Analyses

All data are presented as mean ± SEM. Student’s t test (two-tailed) was used for two group experiments, while one-way ANOVA was used for more that 2 groups comparisons, followed by Tukey’s post-hoc comparisons, unless otherwise stated in figure legends. Asterisks denote p values < 0.05. In bar graphs with more than two conditions, the upper lines whether the indicated the pairwise comparison between the connected bars is significant (*p < 0.05) or not significant (n.s.). When the assumptions for a t-test were not met, a non-parametric Mann-Whitney U test was used, as in data from panel B of Figure S4.

### Data Availability

The datasets generated during and/or analysed during the current study are available from the corresponding author on reasonable request.

## Electronic supplementary material

Supplementary information
