# Author Correction: Remodeling of the Actin/Spectrin Membrane-associated Periodic Skeleton, Growth Cone Collapse and F-Actin Decrease during Axonal Degeneration

**DOI:** 10.1038/s41598-018-23781-w

**Published:** 2018-04-12

**Authors:** Nicolas Unsain, Martin D. Bordenave, Gaby F. Martinez, Sami Jalil, Catalina von Bilderling, Federico M. Barabas, Luciano A. Masullo, Aaron D. Johnstone, Philip A. Barker, Mariano Bisbal, Fernando D. Stefani, Alfredo O. Cáceres

**Affiliations:** 1 Instituto de Investigación Médica Mercedes y Martín Ferreyra, INIMEC-Consejo Nacional de Investigaciones Científicas y Técnicas (CONICET), UNC, Friuli, 2434 – 5016, Córdoba, Argentina; 2 Universidad Nacional de Córdoba, Córdoba, Argentina; 3 Centro de Investigaciones en Bionanociencias (CIBION)-CONICET, Buenos Aires, Argentina; 4 Departamento de Física, Facultad de Ciencias Exactas y Naturales, Universidad de Buenos Aires, Buenos Aires, Argentina; 5 Department of Biology, University of British Columbia, Kelowna, BC Canada; 6 Montreal Neurological Institute, McGill University, Montreal, Canada; 7 Instituto Universitario Ciencias Biomédicas de Córdoba (IUCBC), Córdoba, Argentina

Correction to: *Scientific Reports* 10.1038/s41598-018-21232-0, published online 14 February 2018

The original version of this Article contained errors in the spelling of the authors Sami Jalil and Mariano Bisbal which were incorrectly given as Jalil Sami and Bisbal Mariano respectively. This has now been corrected in the PDF and HTML versions of the Article, and in the accompanying supplementary material.

