# Supplementary material for: Remodeling of the Actin/Spectrin Membrane-associated Periodic Skeleton, Growth Cone Collapse and F-Actin Decrease during Axonal Degeneration
Source: Sci Rep. 2018 Feb 14;8:3007. doi: 10.1038/s41598-018-21232-0 (PMC5812996; doi:10.1038/s41598-018-21232-0)
Supplement: Supplementary file 1 — Supplementary information [file 41598_2018_21232_MOESM1_ESM.pdf]

Supplementary Information

Title

Remodeling of the Actin/Spectrin Membrane-associated Periodic Skeleton, Growth Cone Collapse and F-Actin Decrease during Axonal Degeneration

Authors

Nicolas Unsain, Martin D. Bordenave, Gaby F. Martinez, Sami Jalil, Catalina von Bilderling, Federico M. Barabas, Luciano A. Masullo, Aaron D. Johnstone, Philip A. Barker, Mariano Bisbal, Fernando D. Stefani & Alfredo O. Cáceres

Supplementary Figure S1

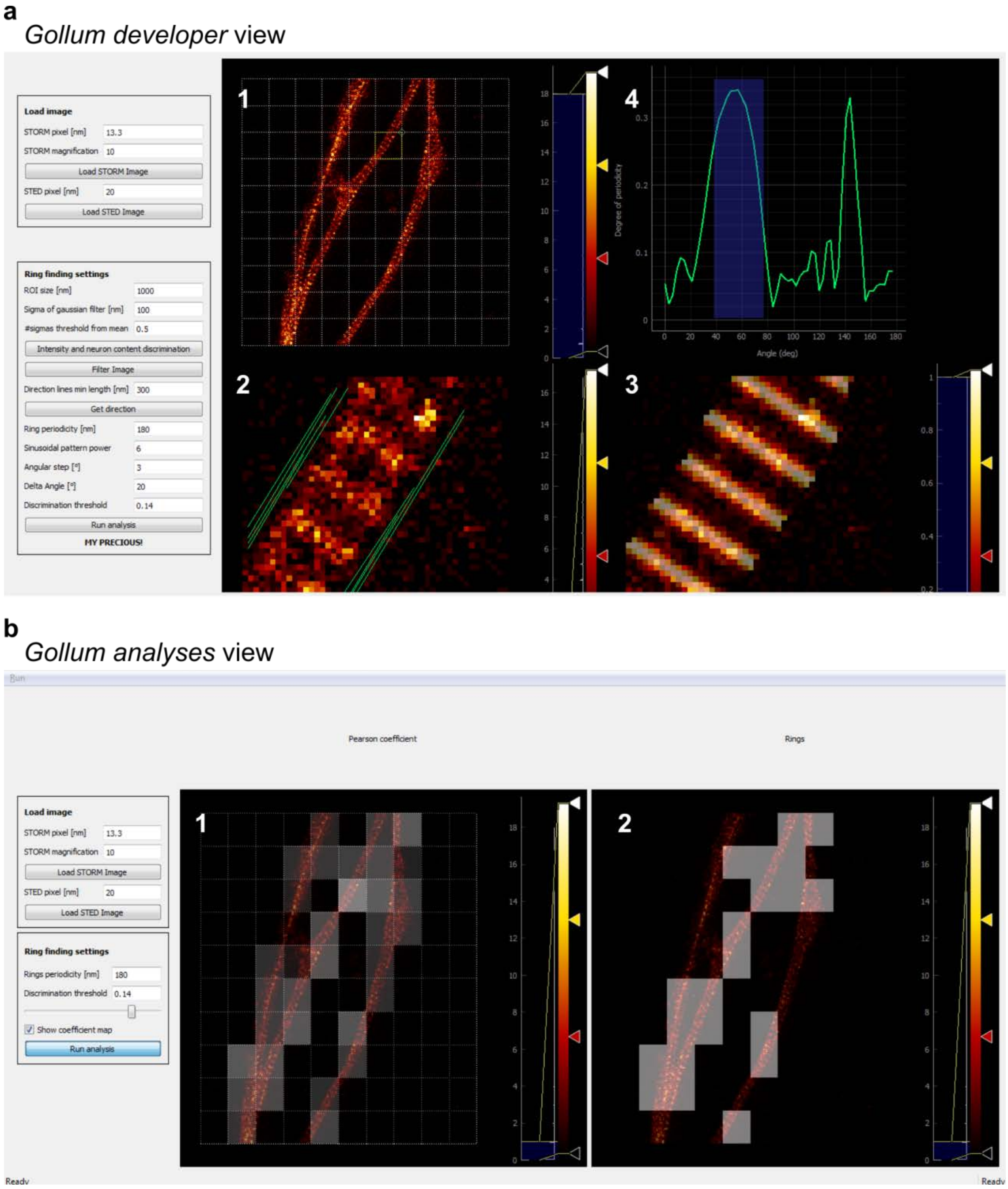

Supplementary Figure S1. Gollum, and image analyses tool to quantitatively characterize periodical structures.(a) Screenshot of Gollum executable in the developer mode. In this mode, the user defines the parameter of the periodical pattern for correlation analysis. In the example, the pattern used in the present report represents an MPS as evidenced by STED

microscopy of βII-spectrin staining. In a loaded image (1), the user selects the sub-region to analyze (2). The software detects the orientation of the axon (2, green lines) and makes the best possible match in phase and angle between the sub-region and the pattern. The correlation values are shown for all the angles (4). The angles range that corresponds to longitudinal direction of the axon are shaded in blue, and is where the correlation maximum is annotated. (b) Screenshot of Gollum executable in the analyses mode. An example image is loaded and the results of the analyses are shown. After axon identification, the tool show all the sub-regions analyzed (1), where the grey values represent the correlation coefficient of each sub-region. The program show the sub-region considered to have a MPS (2) according to the correlation coefficient defined by the user. In batch analyses, the software retrieves a file with the correlation coefficients of all sub-regions analyzed.

Supplementary Figure S2

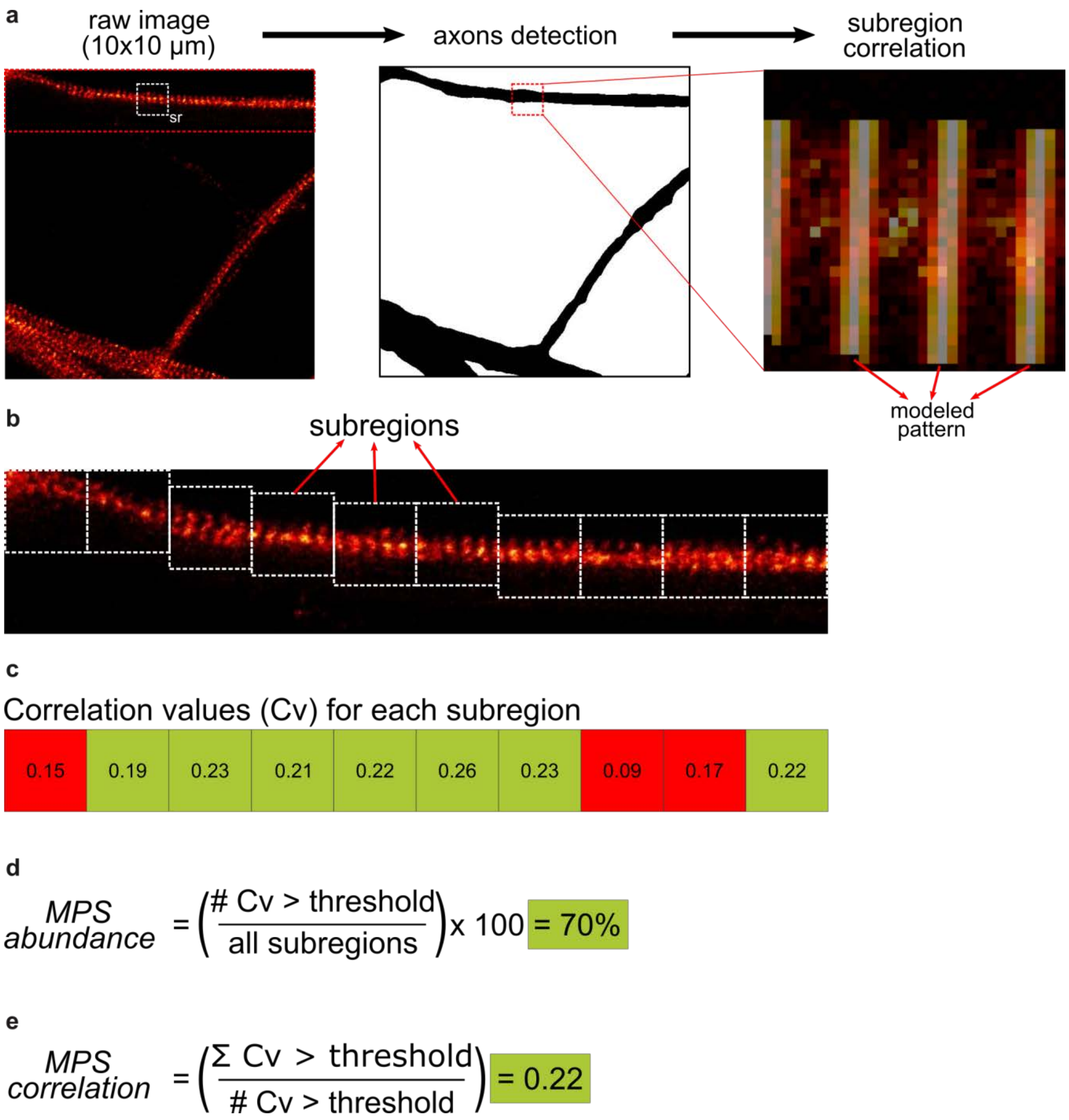

Supplementary Figure S2. Gollum image analyses and results.(a) Flow of image processing. A given sub-region within a detected axon is correlated with a user-defined periodical pattern, in this case a model of the MPS as can be seen by STED nanoscopy of βII-spectrin staining. (b,c) The software scans 1 μm x 1μm consecutive square sub-regions (b), and for each of them retrieves the correlation coefficient (c). According to the defined correlation threshold for an MPS, sub-regions in red are considered not to have an MPS (under 0.17), whereas the green

sub-regions have an MPS.(d) MPS abundance then is defined for a group of images, as the percentage of sub-regions with correlation values above the defined threshold. In the example, being 70%. (e) MPS correlation is defined for a group of images, as the mean correlation value of sub-region above the defined threshold, and is a useful indicator of the level of organization of the periotic structure. In the example, the result is 0.22.

Supplementary Figure S3

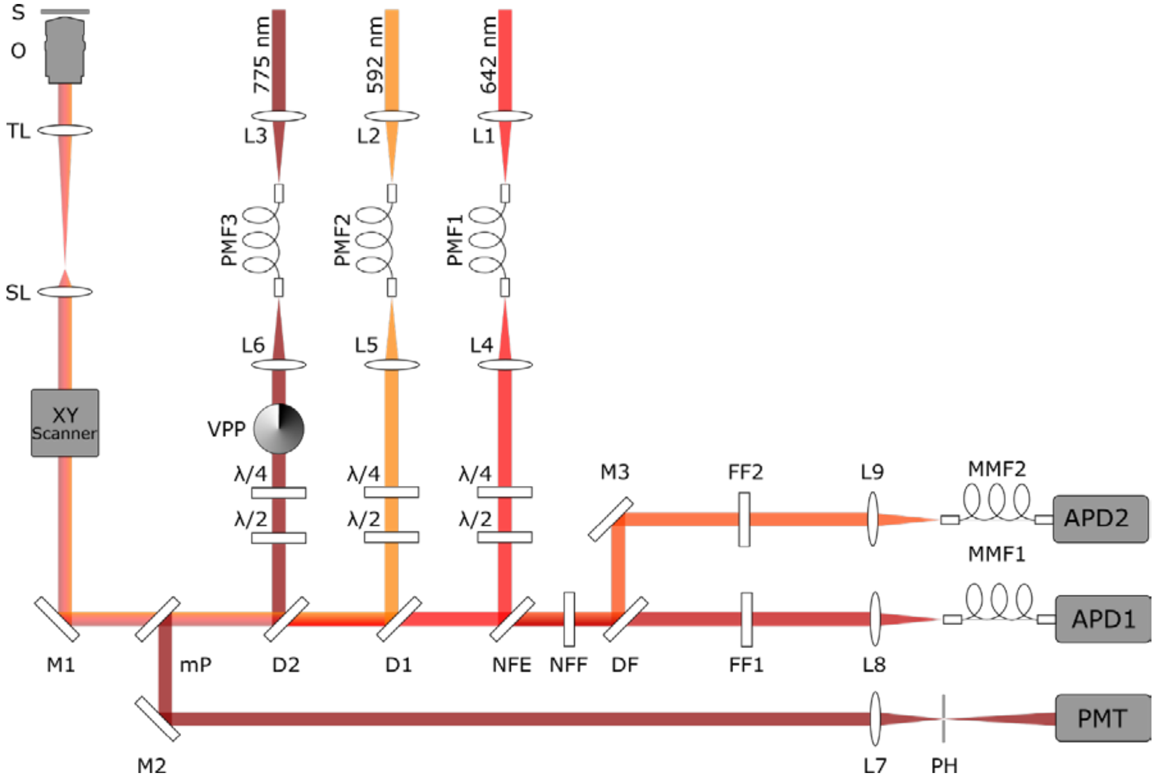

Supplementary Figure S3. Schematical representation of the home built STED microscope used in the study. Refer to the details in the Methods section.
